# Core–shell silica@Cu_*x*_ZnAl LDH catalysts for efficient CO_2_ hydrogenation to methanol†

**DOI:** 10.1039/d3sc02205f

**Published:** 2023-09-01

**Authors:** Meng Lyu, Jianwei Zheng, Claire Coulthard, Jing Ren, Yufei Zhao, Shik Chi Edman Tsang, Chunping Chen, Dermot O'Hare

**Affiliations:** a Chemistry Research Laboratory, Department of Chemistry, University of Oxford 12 Mansfield Road Oxford OX1 3TA UK chunping.chen@chem.ox.ac.uk dermot.ohare@chem.ox.ac.uk +44(0)1865 272686; b Wolfson Catalysis Centre, Department of Chemistry, University of Oxford Oxford OX1 3QR UK; c State Key Laboratory of Chemical Resource Engineering, Beijing University of Chemical Technology 100029 Beijing P. R. China

## Abstract

The efficient production of methanol by reduction of CO_2_ using green hydrogen is a promising strategy from both a green chemistry and a carbon net zero perspective. Herein, we report the synthesis of well-dispersed core–shell catalyst precursors using silica@Cu_*x*_ZnAl-LDHs that can convert CO_2_ to methanol. The catalyst precursors can be formed using either a commercially available silica (ES757) or a mesoporous silica (*e.g.* MCM-48). These hybrid materials show significantly enhanced catalytic performance compared to the equivalent unsupported Cu_*x*_ZnAl LDH precursor. Space-time yields of up to 0.7 g_MeOH_ g_cat_^−1^ h^−1^ under mild operating conditions were observed.

## Abstract 2

The efficient production of methanol by reduction of CO_2_ using green hydrogen is a promising strategy from both a green chemistry and a carbon net zero perspective.

## Graphical abstract

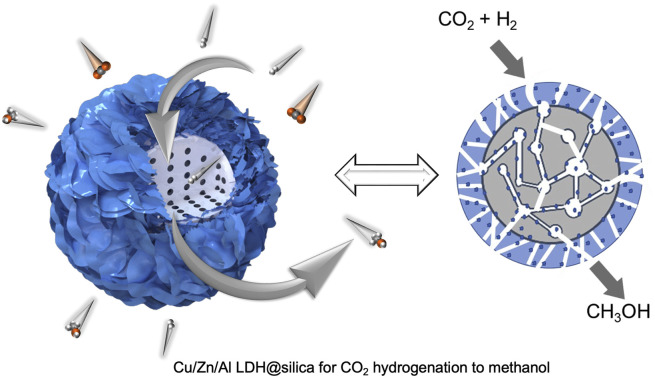

To reverse the rapid increase in atmosphere CO_2_ concentrations it is both urgent and critical we develop efficient strategies to reduce carbon emissions and reach a carbon net zero goal. To date, prodigious efforts have been devoted to developing alternative renewable non-fossil fuel energy sources, as well as CO_2_ capture and utilization strategies. Some of the most important approaches involve the conversion of CO_2_ into liquid hydrocarbons, formic acid, and methanol.^1^ In particular, hydrogenation of CO_2_ to methanol using renewably sourced hydrogen will be strategically a very important component in the portfolio of processes to reach both our emission and decarbonisation targets.^2–4^ Today, hydrogen can be produced from sustainable resources by utilising hydropower, solar energy, and biomass, thus offering the potential for a green “methanol economy”.^5^

Cu_*x*_Zn_*y*_AlO_*z*_ based-catalysts are some of the most widely used catalysts for methanol synthesis from CO_2_.^6,7^ Metallic Cu nanoparticles are generally recognised as the active species, while ZnO and Al_2_O_3_ act as both electronic and geometric promoters for Cu. Several synthetic methods have been developed to assemble these compositions.^6–8^ Layered double hydroxides (LDHs) are a family of anionic 2D layered materials with the general formula, 
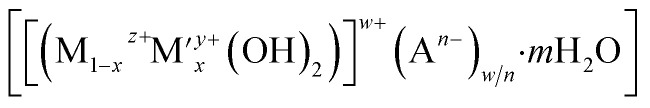
, where M and M′ are typically divalent and trivalent metal cations, octahedrally coordinated by hydroxyl groups and A^*n*−^ represents the charge-compensating intercalated anion.^8^ Cu^2+^, Zn^2+^ and Al^3+^ cations can be incorporated into the LDH structure and used as precursors to Cu_*x*_Zn_*y*_AlO_*z*_ catalysts for methanol synthesis. To obtain a robust catalyst exhibiting high activity, selectivity and lifetime for CO_2_ hydrogenation, two main strategies have been explored for Cu_*x*_Zn_*y*_AlO_*z*_ catalysts derived from LDHs; (i) incorporation of elemental promoters such as Y, Zr, Ga into the Cu_*x*_Zn_*y*_Al LDH;^9–11^ and (ii) exfoliation and dispersion of the LDH layers using the aqueous miscible organic solvent treatment (AMOST) method.^8^ These strategies strive to generate high surface area metallic Cu and also inhibit the Cu nanoparticles from sintering as it is generally accepted that the catalytic activity is a function of exposed metallic Cu surface area to volume ratio. In practise, unsupported Cu_*x*_Zn_*y*_Al LDH platelets tend to stack/aggregate together and following calcination and reduction this results in a decrease in the exposed metallic Cu surface area to volume ratio which limits subsequent catalytic activity.

Herein, we report a new catalyst strategy by synthesising Cu_*x*_ZnAl–CO_3_ LDH precursors supported on a both commercially available silica (ES757) and some mesoporous silica (*e.g.* MCM48, SBA-16) cores, to form an SiO_2_@Cu_*x*_ZnAl LDH core@shell structure. The core@shell catalyst precursors formed using a commercially available silica (ES757) as the core show significantly enhanced catalytic performance compared to the equivalent unsupported Cu_*x*_ZnAl LDH precursor. Using the larger pore volume core silica such as MCM48 provides even further improvements in catalytic performance. The general synthesis procedure of core–shell catalyst is shown in Fig. 1a using ES757 as an exemplar core. ES757 is initially dispersed in Na_2_CO_3_ solution, then Cu_*x*_ZnAl–CO_3_ LDH is generated using an *in situ* co-precipitation approach, the Cu_*x*_ZnAl–CO_3_ LDH nucleates and then grows on both the external and internal surfaces of the ES757 particles to form ES757@Cu_*x*_ZnAl LDH. Calcination at 330 °C for 4 h produces ES757@Cu_*x*_ZnAl LDO, in which the LDH shell is transformed into uniform mixed metal oxide, often referred to as layered double oxide (LDO). The final active catalyst is prepared by reduction of ES757@Cu_*x*_ZnAl LDO under a dilute hydrogen atmosphere (5% H_2_/N_2_) at 290 °C for 2 h. We refer to the finished active catalyst as ES757@Cu_*x*_.

**Fig. 1:**
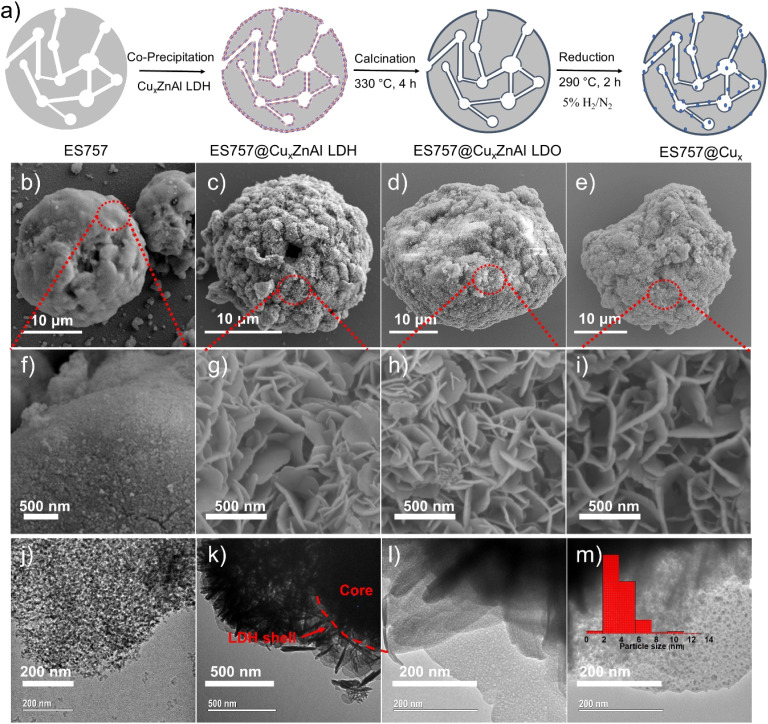
(a) Synthesis scheme of Cu nanoparticles from ES757@Cu_*x*_ZnAl LDH. SEM images of (b and f) ES757; (c and g) ES757@Cu_1.3_ZnAl LDH; (d and h) ES757@Cu_1.3_ZnAl LDO and (e and i) ES757@Cu_1.3_. TEM images of (j) ES757; (k) ES757@Cu_1.3_ZnAl LDH; (l) shell of ES757@Cu_1.3_ZnAl LDO; (m) shell of ES757@Cu_1.3_.

As shown in Fig. 1b, ES757 is supplied as large spherical agglomerates (*ca.* 25 μm diameter) with a relatively smooth surface (Fig. 1f and S1a†). The spherical agglomerates are decorated with a number of tiny silica nanoparticles (*ca.* 10–20 nm diameter) as indicated in Fig. 1j. When Cu_1.3_ZnAl LDH is carefully co-precipitated in the presence of a suspension of ES757 the LDH nucleates and grows from the silica surface. For the LDH coating, we observe a rosette platelet morphology with individual platelets between 300–500 nm (Fig. S2a and d†). In our optimised synthesis conditions, the LDH platelets cover the entire surface, the LDH platelets grow hierarchically from the ES757 surface forming uniform core–shell particles (Fig. 1c and k) and a honeycomb surface (Fig. 1g and S1b†) texture. As a control, a physical mixture of ES757 and Cu_1.3_ZnAl–CO_3_ LDH (prepared by manually mixing the two solids in 40 : 60 the weight ratio) shows separated particles that retain their individual morphological features (Fig. S1c and d†). After calcination at 330 °C, the unsupported Cu_1.3_ZnAl LDO platelets condense and aggregate together (Fig. S2b and e†). In contrast, calcination of ES757@Cu_1.3_ZnAl LDH produces LDO platelets still immobilised on the ES757 and retaining their honeycomb structure (Fig. 1d and h). Further reduction by hydrogen, produces metallic Cu nanoparticles embedded on the LDO matrix which we refer to as ES757@Cu_1.3_.^8,12^ In ES757@Cu_1.3_, the metallic Cu(0) nanoparticles are well dispersed with size distribution of 3.9 ± 1.6 nm (Fig. 1m). In comparison, hydrogen reduction of the unsupported Cu_1.3_ZnAl LDH (named as Cu_1.3_) produces nanoplatelets stacked together (Fig. S2c and f†), with lower exposed metallic Cu(0). The electron mapping images (Fig. 2a–e) of ES757@Cu_1.3_ZnAl LDH demonstrate that Cu, Zn and Al from the Cu_1.3_ZnAl LDH and Si from ES757 are homogeneously dispersed across the whole spherical particle.

**Fig. 2:**
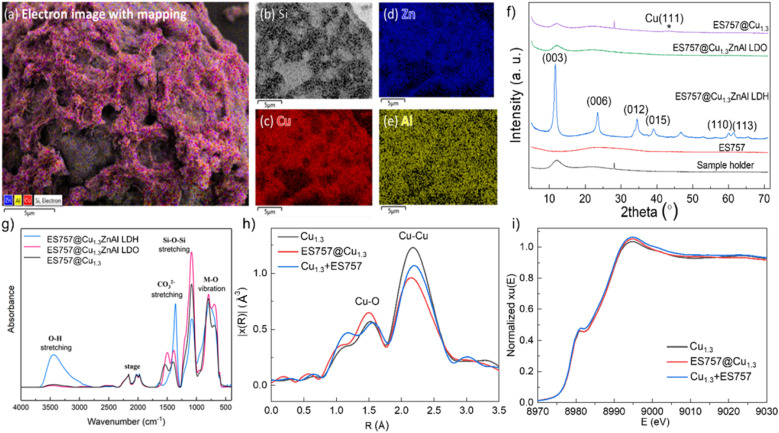
Structural evolution during the preparation of ES757@Cu_1.3_. (a) Electron image with Zn, Al, Cu, Si element mapping; (b) Si; (c) Cu; (d) Zn; (e) Al of ES757@Cu_1.3_ZnAl LDH; (f) powder X-ray diffraction patterns; (g) FTIR spectra; (h) Fourier transformed magnitudes of the experimental Cu K-edge EXAFS spectra; (i) normalised Cu K-edge XANES spectra.

Powder X-ray diffraction data (Fig. 2f) for ES757 exhibits a broad feature between 2*θ* = 15–35° typical of an amorphous silica-based material. The XRD of ES757@Cu_1.3_ZnAl LDH shows this amorphous background superimposed with the characteristic (00*l*) and (110) Bragg reflections associated with the crystalline LDH layer. The *d*-spacing of the (003) Bragg reflection of ES757@Cu_1.3_ZnAl LDH at 2*θ* = 7.58 Å, indicates a carbonate-intercalated LDH.^13^ After calcination, the characteristic LDH Bragg reflections disappear and they are replaced by a broad weak Bragg reflection at 2*θ* ≈ 38° (Fig. 2f and S8b†). This feature can be assigned to the formation of copper oxide. Following reduction by hydrogen, a very weak Bragg reflection at 2*θ* ≈ 43° corresponding to (111) reflection from metallic copper is observed.

The FTIR spectrum (Fig. 2g) of ES757@Cu_1.3_ZnAl LDH exhibits both the characteristic IR vibrations bands of ES757 (the Si–O stretching at 1050 cm^−1^) and LDH shell (the metal–oxygen (M–O) vibration at 750 cm^−1^ and the O–H stretching of M-OH and water at 3440 cm^−1^, and water bending at 1600 cm^−1^).^13–15^ Upon calcination, the water bending absorbance (1600 cm^−1^) disappears and the O–H stretching band (3500 cm^−1^) reduces in intensity due to dehydration and dihydroxylation of the LDH shell. A sharp band at 1350 cm^−1^ in ES757@Cu_1.3_ZnAl LDH confirmed the presence of intercalated carbonate ions.^16,17,19^

The chemical composition of both unsupported Cu_1.3_ZnAl LDH and ES757@Cu_1.3_ZnAl LDH were investigated using elemental analyses and thermogravimetric analysis (TGA). The Cu/Al ratio of both pristine Cu_1.3_ZnAl LDH and the core–shell materials was determined by inductively coupled plasma optical emission spectrometry (ICP-OES). The data agree with the predicted values (Table S1†). The TGA data (Fig. S3†) for ES757@Cu_1.3_ZnAl LDH show the typical weight loss stages for an LDH, we can also use the TGA data to determine the ES757 : LDH ratio in the core–shell materials as 40 : 60, again in agreement with the predicted value. The formula of Cu_1.3_ZnAl LDH and ES757@Cu_1.3_ZnAl LDH can be obtained from elemental analysis and TGA, which are [Cu_1.31_Zn_0.93_Al(OH)_6.49_(CO_3_)_0.49_·4H_2_O] and [SiO_2_]_3.85_[Cu_1.29_Zn_1.03_Al(OH)_6.64_(CO_3_)_0.50_·1.49H_2_O]_1.05_, respectively.^10^

N_2_ adsorption and desorption isotherms were used to investigate the porosity of the samples. The specific surface areas (Fig. S5a†) of ES757 and Cu_1.3_ZnAl LDH using BET analysis are 273 m^2^ g^−1^ and 48 m^2^ g^−1^, respectively. Upon forming ES757@Cu_1.3_ZnAl LDH, the BET surface area becomes 189 m^2^ g^−1^, which is still higher than the theoretical surface area (138 m^2^ g^−1^) considering the 40 : 60 weight ratio (ES757 : LDH). As shown in Fig. S4a,† Cu_1.3_ZnAl LDH exhibits a type II isotherm with a H3 loop, indicating the agglomerate of platelets with slit-like pores. The BJH derived pore size distribution for Cu_1.3_ZnAl LDH (Fig. S4b†) shows a wide pore size distribution with no clear maxima due to intense interplatelet aggregation.^20^ ES757 presents a type II isotherm with a H2b hysteresis loop, indicating that it is a mesoporous material in which the pore body is slightly larger than the pore neck. The pores in ES757 have a diameter in the range of 25–125 nm. The N_2_ adsorption and desorption isotherms of ES757@Cu_1.3_ZnAl LDH are identical to that of ES757, demonstrating the overall pore structure is retained following the formation of the core–shell structure. The pore size of ES757@Cu_1.3_ZnAl LDH is smaller and in a narrower range (Fig. S4b†), indicating some Cu_1.3_ZnAl LDH may slightly block some of the pore openings in ES757. These observations indicate that immobilising an LDH on a porous substrate can avoid the aggregation of LDH platelets and exposes more surface area. A similar trend was found in total pore volume (Fig. S5b†). Finally, calcination followed by reduction by hydrogen did not cause significant changes to the overall structural morphology the hybrid materials (Fig. S4†). We observe a slight increase in the specific surface area after calcination (Fig. S5†) which is due to the formation of amorphous porous network for Cu_1.3_ZnAl LDO.

The X-ray absorption near-edge structure (XANES) spectroscopy and extended X-ray absorption fine structure (EXAFS) measurements were measured to probe the nature of Cu species in three catalysts prepared by calcination and reduction of (i) Cu_1.3_ZnAl LDH, (ii) ES757@Cu_1.3_ZnAl LDH and (iii) Cu_1.3_ + ES757 (a physical mixture of Cu_1.3_ZnAl LDH and ES757). All the samples show two characteristic features in the first shell (Fig. 2h). The most noticeable scattering was observed at 2.2 Å which is ascribed to the Cu–Cu separation. A weak feature at 1.7 Å is assigned to Cu–O. According to the fitting of the EXAFS (Fig. S6, S7 and Table S3†), ES757@Cu_1.3_ has the lowest number of Cu–Cu nearest neighbour interactions, indicating ES757@Cu_1.3_ has the best dispersion compared to Cu_1.3_ and Cu_1.3_ + ES757. XANES spectra in Fig. 2i shows that the Cu K edge feature falls at the highest energy in ES757@Cu_1.3_ which suggests Cu is more electronic deficient (Cu^*δ*+^), these data are consistent with formation of well dispersed Cu nanoparticles in contact to the support through Cu–O interactions.

The catalytic performance of Cu_1.3_, ES757@Cu_1.3_ and Cu_1.3_ + ES757 for CO_2_ hydrogenation to methanol was evaluated. As shown in Fig. 3a, ES757@Cu_1.3_ and Cu_1.3_ + ES757 exhibit higher CO_2_ conversion (23% and 26%, respectively) than that of Cu_1.3_ (16%). It is believed that the introduction of ES757 has a synergistic effect benefiting mass transfer and therefore promote CO_2_ conversion.^21^ Noteworthy, the CO_2_ conversions of ES757@Cu_1.3_ and Cu_1.3_ + ES757 are approaching the equilibrium value under our catalytic testing conditions.^22^ More importantly, ES757@Cu_1.3_ shows the highest methanol selectivity of 48% (the only other C-product detected was CO) which is significantly higher than that of Cu_1.3_ + ES757 (28%). The particle sizes arelisted in Table S2,† it indicates high dispersion of Cu species. It should be noted that the particle sizes derived from TEM are a bit larger than those from XRD. We believe that this may arise from the high copper dispersion as evidenced from EXAFS and the measured copper surface area (*S*_Cu_). It is widely accepted that stable, well-dispersed metallic copper with a large *S*_Cu_ is linked to overall methanol productivity.^23^ N_2_O chemisorption was used to determine the *S*_Cu_. The copper dispersion^18^ is then determined from exposed copper surface (*S*_Cu_)^24,25^ and total copper loading. We found that both the copper dispersion and exposed copper surface area of ES757@Cu_1.3_ (25%, 32.14 m^2^ g_catalyst_^−1^) is much higher than that of Cu_1.3_ (13.57%, 23.05 m^2^ g_catalyst_^−1^) and Cu_1.3_ + ES757 (1.88%, 17.07 m^2^ g_catalyst_^−1^) (Table S2†). It is apparent that a catalyst prepared without a support or simply mixing the catalyst with porous silica cannot avoid the aggregation between LDH platelets, leading to much lower exposed copper surface. Immobilising and orienting the LDH catalyst precursor on the porous silica creates a highly dispersed and exposed surface bound active metallic copper sites, resulting in the superior CO_2_ conversion and methanol selectivity. This enables ES757@Cu_1.3_ to exhibit the highest space-time yield for methanol (STY_MeOH_ 0.64 g_methanol_ g_cat_^−1^ h^−1^) among these three catalysts.

**Fig. 3:**
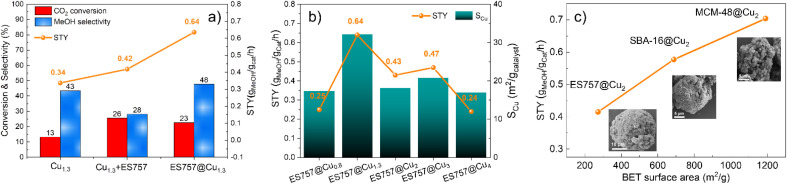
Catalytic performance and structure relationship. The effect of (a) core–shell structure, (b) Cu loading and Cu surface area and (c) specific surface area (insets are the HRSEM of core–shells) on the catalytic performance. Reaction condition: temperature = 270 °C, pressure = 45 bar, H_2_ : CO_2_ (molar) = 3 : 1, WHSV = 18 000 mL g_catalyst_^−1^ h^−1^.

We further studied the effects of copper dispersion and *S*_Cu_ by preparing a series of ES757@Cu_*x*_ZnAl–CO_3_–LDH core–shells with different copper loadings. The core–shells were prepared using the same procedure as ES757@Cu_1.3_ZnAl LDH, the copper content was controlled by the ratio of metal salts used in the co-precipitation step. The series of precursors, ES757@Cu_*x*_ZnAl–CO_3_–LDH (*x* = 0.8–4) were prepared and following calcination and hydrogen reduction were named ES757@Cu_*x*_ (*x* = 0.8–4). HR-SEM images (Fig. S8†) confirm the formation of core–shell structures and the powder XRD data (Fig. S9a†) indicate no other crystalline impurities. Following calcination, ES757@Cu_*x*_ZnAl LDOs do not present any LDH Bragg reflections. Following hydrogen reduction, the intensity of metallic Cu 111 Bragg reflection increases in intensity with increasing copper loading. The copper loading, dispersion, *S*_Cu_ and the catalytic performance (turnover frequency (TOF) and space time yield STY) are summarised in Table S2.† The relationship between STY_MeOH_ and *S*_Cu_ is shown in Fig. 3b. It is found that both copper dispersion and *S*_Cu_ increase with increasing copper loading, reaching a maximum at ES757@Cu_1.3_. Further increasing the copper loading results in the decrease of copper dispersion and *S*_Cu_ due to the sintering of the metallic Cu nanoparticles at high loadings. The STY_MeOH_ follows the same trend as copper dispersion and *S*_Cu_. ES757@Cu_1.3_ with the highest copper dispersion and largest *S*_Cu_ exhibits the highest STY_MeOH_ among all core–shell materials we have studied.

The catalytic effects of other porous silica cores were explored. We have use of SBA-16 and MCM-48 with specific surface areas of 688 and 1186 m^2^ g^−1^ respectively. The synthesis method is the same as that of ES757@Cu_*x*_ZnAl LDHs, the copper loading was kept constant at Cu : Zn : Al = 2 : 1 : 1. The calcined and reduced catalysts derived from SBA-16@Cu_2_ZnAl–CO_3_ LDH and MCM-48@Cu_2_ZnAl–CO_3_ LDH were named SBA-16@Cu_2_ and MCM-48@Cu_2_ respectively. As shown in Fig. 3c (inset HRSEM) and Fig. S9–14,† these samples formed the similar core–shell structures as that of ES757@Cu_1.3_ZnAl LDH. A uniform distribution of Cu, Zn and Al is observed over the MCM-48 and SBA-16 particles as evidenced by EDX mapping. The influence of the specific surface areas of these cores on the catalytic performance of the final catalyst can be seen in Fig. 3c. Generally, the catalysts with higher surface area exhibit higher STY_MeOH_. It is possible that copper may distribute more evenly over the surface when employing a silica core with a higher surface area.^21,26^

The catalytic performance of the core–shell catalysts developed in this work were compared with those of an commercial Cu/ZnO/Al_2_O_3_ catalyst and our best Cu-catalyst (Cu_1.3_ZnGaO_*y*_) derived from the exfoliated Cu_1.3_ZnGa LDH precursor, under the same catalytic conditions.^8^ As shown in Table S3†, both ES757@Cu_1.3_ and MCM-48@Cu_2_ exhibit higher CO_2_ conversion and methanol selectivity. These catalysts deliver superior STY_MeOH_ of 0.64 and 0.7 g_methanol_ g_cat_^−1^ h^−1^, respectively more than Cu_1.3_ZnGa (STY_MeOH_ = 0.59 g_methanol_ g_cat_^−1^ h^−1^), delivering nearly twice the commercial catalyst (STY_MeOH_ = 0.38 g_methanol_ g_cat_^−1^ h^−1^) under our testing conditions. It is also important to note that the copper loadings in our core–shell catalysts is typically 19.81–24.5 wt%, significantly lower than that of Cu_1.3_ZnGa (33.5 wt%) and commercial catalyst (50.0 wt%). Therefore, on a per Cu atom basis these core–shell catalysts contain significantly more active individual catalytic sites.

## Conclusion

We have developed effective syntheses of a series of core–shell catalysts using micro- and mesoporous silica cores and a Cu_*x*_ZnAl LDH as a functional shell. The silica cores have excellent coverage by the LDHs platelets and are robust to calcination. Hydrogen reduction of the silica@Cu_*x*_ZnAl LDO precursors produces robust and highly dispersed metallic Cu nanoparticles with high *S*_Cu_. We believe this strategy delivers an effective pathway to prevent the aggregation of the active catalytic sites under catalytic conditions. ES757@Cu_1.3_ was found as our best performing catalyst to date among the ES757@Cu_*x*_ family, although this can be further improved by using a higher surface area silica core (*e.g.* MCM-48). The STY_MeOH_ for these materials places them as some of the best performing catalytic systems for CO_2_ hydrogenation to MeOH, especially if you normalise on a per Cu basis.

## Data availability

Experimental procedures, and spectroscopic data can be found in the ESI.†

## Author contributions

Meng Lyu and Jianwei Zheng, performed the synthetic and catalysis experimental work; Claire Coulthard assisted with the electron microscopy studies; Ren Jing, and Yufei Zhao recorded and interpreted the X-ray absorption analysis; Shik Chi Edman Tsang, Chunping Chen, and Dermot O'Hare conceptualised the research, acquired funding, and supervised the work; all authors revised and edited the manuscript. All authors have read and agreed to the published version of the manuscript.

## Conflicts of interest

The authors declare no competing financial interests.

## Supplementary Material

SC-014-D3SC02205F-s001

## References

[cit1] Ye R.-P., Ding J., Gong W., Argyle M. D., Zhong Q., Wang Y., Russell C. K., Xu Z., Russell A. G., Li Q. (2019). Nat. Commun..

[cit2] Kattel S., Ramírez P. J., Chen J. G., Rodriguez J. A., Liu P. (2017). Science.

[cit3] Bahruji H., Bowker M., Hutchings G., Dimitratos N., Wells P., Gibson E., Jones W., Brookes C., Morgan D., Lalev G. (2016). J. Catal..

[cit4] Martin O., Martín A. J., Mondelli C., Mitchell S., Segawa T. F., Hauert R., Drouilly C., Curulla-Ferré D., Pérez-Ramírez J. (2016). Angew. Chem., Int. Ed..

[cit5] Stephan D. W. (2013). Nature.

[cit6] Jiang X., Nie X., Guo X., Song C., Chen J. G. (2020). Chem. Rev..

[cit7] Meshkini F., Taghizadeh M., Bahmani M. (2010). Fuel.

[cit8] Li M. M.-J., Chen C., Ayvalı T. c. e., Suo H., Zheng J., Teixeira I. F., Ye L., Zou H., O'Hare D., Tsang S. C. E. (2018). ACS Catal..

[cit9] Gao P., Li F., Xiao F., Zhao N., Wei W., Zhong L., Sun Y. (2012). Catal. Today.

[cit10] Gao P., Li F., Zhao N., Xiao F., Wei W., Zhong L., Sun Y. (2013). Appl. Catal., A.

[cit11] Kühl S., Schumann J., Kasatkin I., Hävecker M., Schlögl R., Behrens M. (2015). Catal. Today.

[cit12] Behrens M., Studt F., Kasatkin I., Kühl S., Hävecker M., Abild-Pedersen F., Zander S., Girgsdies F., Kurr P., Kniep B.-L. (2012). Science.

[cit13] Cavani F., Trifiro F., Vaccari A. (1991). Catal. Today.

[cit14] Ye R.-P., Lin L., Chen C.-C., Yang J.-X., Li F., Zhang X., Li D.-J., Qin Y.-Y., Zhou Z., Yao Y.-G. (2018). ACS Catal..

[cit15] Akpotu S. O., Moodley B. (2018). J. Mol. Liq..

[cit16] Smyrnioti M., Tampaxis C., Steriotis T., Ioannides T. (2020). Catal. Today.

[cit17] Lavalley J. (1996). Catal. Today.

[cit18] Zeng R.-C., Li X.-T., Liu Z.-G., Zhang F., Li S.-Q., Cui H.-Z. (2015). Front. Mater. Sci..

[cit19] Cardinale A. M., Carbone C., Consani S., Fortunato M., Parodi N. (2020). Crystals.

[cit20] Chen C., Wangriya A., Buffet J.-C., O'Hare D. (2015). Dalton Trans..

[cit21] Koh M. K., Khavarian M., Chai S. P., Mohamed A. R. (2018). Int. J. Hydrogen Energy.

[cit22] Stangeland K., Li H., Yu Z. (2018). Ind. Eng. Chem. Res..

[cit23] Sagar G. V., Rao P. V. R., Srikanth C. S., Chary K. V. (2006). J. Phys. Chem. B.

[cit24] Brands D. S., Poels E. K., Krieger T. A., Makarova O. V., Weber C., Veer S., Bliek A. (1996). Catal. Lett..

[cit25] Robinson W., Mol J. (1990). Appl. Catal..

[cit26] Murthy P. S., Liang W., Jiang Y., Huang J. (2021). Energy Fuels.

