# Supplementary material for: Core–shell silica@CuxZnAl LDH catalysts for efficient CO2 hydrogenation to methanol
Source: Chem Sci. 2023 Sep 1;14(36):9814–9. doi: 10.1039/d3sc02205f (PMC10510760; doi:10.1039/d3sc02205f)
Supplement: SC-014-D3SC02205F-s001 [file SC-014-D3SC02205F-s001.pdf]

Electronic Supplementary Material (ESI) for Chemical Science.
This journal is © The Royal Society of Chemistry 2023

Supplementary material

Core-shell silica@Cu_xZnAl LDH catalysts for efficient CO_2 hydrogenation to methanol †

Meng Lyu[ǂa], Jianwei Zheng[ǂb], Claire Coulthard[a], Ren Jing[c], Yufei Zhao[c], Shik Chi Edman Tsang[b], Chunping Chen*[a], and Dermot O'Hare*[a]

Contents

1. Experimental section

2. Supporting figures

3. Supporting tables

1. Experimental section

1.1 Synthesis

1.1.1 Synthesis of $Cu_{1.3}$ZnAl LDH, LDO and $Cu_{1.3}$ catalyst.

$Cu_{1.3}$ZnAl LDH was prepared using a co-precipitation method. Na_2CO_3 (0.68 g, 6.41 mmol) was added to de-ionised (DI) water (24.00 mL) with stirring (stirring speed 500 rpm) to prepare solution A. The pH value of solution A was adjusted to 9 with 10 wt% nitric acid. An aqueous solution (64 mL) of $Cu(NO_3)_2{\cdot}3H_2O$ (1.22 g, 5.05 mmol), $Zn(NO_3)_2{\cdot}6H_2O$ (1.16 g, 3.89 mmol) and $Al(NO_3)_3{\cdot}9H_2O$ (1.46 g, 3.89 mmol) was added to solution A within eighty minutes (dropping rate of metal salt solution is 49 mL/min). The pH value of the reacting mixture was kept at 9 using 4 M NaOH aqueous solution. The resulting mixture was aged for one hour under stirring (stirring speed 500 rpm) after the completion of addition. The product was collected by Buchner filtration and washed with water till the pH of filtrate was equal to 7 (approximately 1.5 L DI water was used) before being dried under vacuum at 30 °C overnight.

$Cu_{1.3}$ZnAl LDO was obtained from the calcination of to afford $Cu_{1.3}$ZnAl LDH at 330 °C for 4 hours at a 2 °C/min ramp rate under constant air flow at 50 mLn/min. During the process, the sample was held at 80 °C and 150 °C for one hour dwell time respectively.

$Cu_{1.3}$ catalyst was prepared by reduction of $Cu_{1.3}$ZnAl LDO at 290 °C for 2 hours at 2 °C/min ramp rate under constant gas flow (5% H_2/N_2) at 50 stp mL/min (stp = standard temperature and pressure: P = 101.3 kPa, T = 298 K).

1.1.2 Synthesis of $mSiO_2$@$Cu_{1.3}$ZnAl LDH, $mSiO_2$@$Cu_{1.3}$ZnAl LDO and $mSiO_2$@$Cu_{1.3}$

Core-shell **$mSiO_2$@$Cu_{1.3}$ZnAl LDH** was prepared using a co-precipitation method. The $mSiO_2$ (ES757, SBA-16 or MCM-48, 0.80 g) was mixed with de-ionised (DI) water (24.00 mL) in beaker (150 mL or 200 mL tall beaker) by thirty-minute sonication. Na_2CO_3 (0.68 g, 6.41 mmol) was then added to the dispersion which was stirred for five minutes (stirring speed 500 rpm). The achieved mixture was named as solution A. The pH value of solution A was adjusted to 9 with 10 wt% nitric acid. An aqueous solution (64 mL) of $Cu(NO_3)_2{\cdot}3H_2O$ (1.22 g, 5.05 mmol), $Zn(NO_3)_2{\cdot}6H_2O$ (1.16 g, 3.89 mmol) and $Al(NO_3)_3{\cdot}9H_2O$ (1.46 g, 3.89 mmol) was added to solution A within eighty minutes (dropping rate of metal salt solution is 49 mL/min). The pH value of the reacting mixture was kept at 9 using 4 M NaOH aqueous solution. The resulting mixture was aged for one hour under stirring (stirring speed 500 rpm) after the completion of addition. The product was collected by Buchner filtration and washed with water till the pH of filtrate was equal to 7 (approximately 1.5 L DI water was used) before being dried under vacuum at 30 °C overnight.

$mSiO_2@Cu_{1.3}ZnAl$ LDO was prepared by the calcination of $mSiO_2@Cu_{1.3}ZnAl$ LDHs at 330 °C for 4 hours at a 2 °C/min ramp rate under constant air flow at 50 mL_n/min. During the process, the sample was held at 80 °C and 150 °C for one hour dwell time respectively.

$mSiO_2@Cu_{1.3}$ was obtained from the reduction of $mSiO_2@Cu_{1.3}ZnAl$ LDO at 290 °C for 2 hours at 2 °C/min ramp rate under constant gas flow (5% H_2/N_2) at 50 stp mL/min (stp = standard temperature and pressure: P = 101.3 kPa, T = 298 K).

1.1.3 Synthesis procedure of ES757@Cu_xZnAl LDH

For preparing core-shells with different copper loading, only the usage of metal salt and the usage of sodium carbonate are changed, the rest procedure remains the same as the synthesis of ES757@$Cu_{1.3}ZnAl$ LDH. The details are listed in the table below.

Sample name	Amount of metal nitrate (g)			Amount of
	$Cu(NO_3)_2)\cdot 3H_2O$	$Zn(NO_3)_2)\cdot 3H_2O$	$Al(NO_3)_3)\cdot 9H_2O$	Na_2CO_3 (g)
ES757@$Cu_{0.8}ZnAl$ LDH	0.885	1.363	1.718	0.680
ES757@Cu_2ZnAl LDH	1.549	0.954	1.203	0.680
ES757@Cu_3ZnAl LDH	1.859	0.763	0.962	0.544
ES757@Cu_4ZnAl LDH	2.066	0.636	0.802	0.450

These composites were activated (calcined and reduced) follow the same procedure as that was used for ES757@$Cu_{1.3}ZnAl$ LDH to produce core-shell ES757@Cu_x catalysts.

1.2 Characterization

Standard powder X-ray diffraction (PXRD) analysis was carried out on a Bruker D8 ECO diffractometer with Cu Kα radiation (45 kV, 40 mA). Signals between 2θ = 3–70° were gathered with step size 0.0298°. Rietveld refinement were performed adopting TOPAS-Academic V6.[1] For lab XRD data, refining crystallite size and microstrain broadening at the same time may lead to an over-parameterisation and end up with meaningless numbers. Therefore, only crystallite size is refined, which is calculated from the integral breadth of peaks. Its value is an underestimation of the average crystallite size; a change of the value indicates a change in the phase crystallinity.

High resolution SEM (HR-SEM) images and EDX was achieved using a Zeiss Merlin high-resolution scanning electron microscope at an accelerating voltage of 5 kV, at DCCEM within the Department of Materials, University of Oxford.

Transmission electron microscopy (TEM) images and EDX were obtained from a JEOL-2100 electron microscope with an accelerating voltage of 200 kV using a single tilt specimen at DCCEM within the Department of Materials, University of Oxford.

Fourier transform Infrared spectroscopy (FTIR) was conducted utilizing a ThermoScientific Nicolet iS5 spectrometer fitted with an iD3 attenuated total reflection stage. Signals between 4000–700 cm^{-1} wavelengths were recorded at 4 cm^{-1} resolution.

Thermogravimetric analysis (TGA) was carried out under a nitrogen atmosphere utilizing Mettler Toledo TGA/DSC 1 system or PerkinElmer TGA 8000. The weight change between 30–800 °C was collected with 5 °C/min ramp rate.

Elemental C, H, N analysis (EA) and **inductively coupled plasma atomic emission spectroscopy (ICP-OES)** were conducted by Dr Nigel Howard at Microanalysis, University of Cambridge.

N_2 adsorption/desorption isotherms of mesoporous (as well as macro-porous) and microporous materials were collected employing Micrometric 3Flex respectively at 77 K. All sample were *ex-situ* degassed on Micromeritics VacPrep overnight before the measurement. ES757@Cu_xZnAl-CO_3-LDH core-shells were *ex-situ* degassed overnight at 110 °C and *in-situ* degassed at 110 °C for 1 hour. ES757@Cu_xZnAlLDOs and ES757@Cu_xZnAl catalysts was *ex-situ* degassed at 35 °C and *in-situ* degassed at 100 °C for 1 hour.

The pore structure is investigated by the N_2 adsorption and desorption isotherm at 77 K. Specific surface area is estimated using the Brauner-Emmett-Teller (BET) method.[2] Pore size distribution within the micropore (< 2 nm) and mesopore (2–50 nm) is calculated by applying an appropriate DFT kernel to the adsorption isotherm. For silica and core-shell composite, the adsorption of N_2 at 77 K on metal oxide with cylindrical pore kernel was selected.[3] For LDH, the adsorption of N_2 at 77 K with slit-pore kernel was adopted.[4] Macropore (> 50 nm) size distribution is determined utilising Barrett-Joyner-Halenda (BJH) method.[2]

Hydrogen temperature programmed reduction (H_2-TPR)

H_2-TPR curves were recorded on a Micromeritics AutoChem II 2920 Chemisorption Analyser equipped with a thermal conductivity detector (TCD). Around 120 mg of calcined core-shell was loaded in a quartz U-tube and purged with He (10 cm^3/min) for 5 minutes at room temperature. This He flow was maintained while the sample was heated to 150 °C (10 °C/min) and hold for 5 minutes to remove moisture and impurities. After cooling to 40 °C, the reduction step started. The sample was heated from 40 to 600 °C at a rate of 5 °C/min under a H_2 flow (10% H_2 in N_2 at 50 cm^3/min) with a sampling rate of 1 measurement per second.

Nitrous oxide (N_2O) reduction of ES757@Cu_xZnAl catalysts

The dispersion (D_{Cu}) and exposed surface area (S_{Cu}) of Cu were determined by dissociative N_2O chemisorption followed by hydrogen pulse reduction. N_2O chemisorption was carried out on a Micromeritics AutoChem II 2920 instrument. Before the measurement, 100 mg of calcined sample was reduced at 290 °C in a 10% H_2/Ar mixture (50 mL/min) for 2 h. After cooling to 60 °C, the sample

was exposed to N_2O (20 mL/min) for 1 h to ensure complete oxidation of surface metallic copper to Cu_2O. Subsequently, the sample was flowed by N_2 (50 mL/min) to remove residual N_2O in the sample tube and physisorbed N_2O on the surface. Finally, a calibrated hydrogen pulse reduction at 290 °C was conducted to determine the amount of surface Cu_2O species. D_{Cu} and S_{Cu} were then calculated by dividing the amount of surface copper by the actual Cu loading determined by ICP-OES.

Extended X-ray absorption fine structure spectroscopy (EXAFS) and X-ray absorption near-edge structure (XANES)

The Cu K-edge XAFS was recorded at the 1W1B beamline of Beijing Synchrotron Radiation Facility at room temperature. A Si(1 1 1) Double Crystal Monochromator was employed to scan the photon energy. The energy resolution ($\Delta E/E$) for the incident X-ray photons was around 2×10^{-4}. Conventional transmission mode was applied for Cu K-edge EXAFS measurements. And the samples were in pellets for the measurements. The EXAFS data analysis was obtained using IFEFFIT 1 with Horae packages 2 (Athena and Artemis). Cu foils was used for calibration as a reference to avoid energy shifts of the samples. And the amplitude parameter (0.87) was obtained from EXAFS data analysis of the foil.

1.3 Catalytic evaluation of CO_2 hydrogenation to methanol

Catalytic tests for CO_2 hydrogenation to methanol were conducted utilizing a tubular fixed bed reaction (12.7 mm outside diameter). Typically, 0.1 g of a ground calcined sample was used, then *in-situ* reduced at 290 °C for 2 hours at a 2 °C/min ramp rate under a constant gas flow (5% H_2/N_2) at 50 stp mL/min. The reduction temperature was optimised at 290 ^{o}C (Table S4). Subsequently, the reactor was pressurised to 4.5 MPa with CO_2/H_2 (volume ratio 1:3) once the sample had cooled to below 50 °C. The activity of the catalysts was determined using a constant flow of a 1:3 CO_2 : H_2 gas mixture through the catalyst bed (30 stp mL/min) at 270 °C. The activity measurements were reported after at minimum of 2 hours on the stream at each reaction temperature. The performance data reported was the average value of the last three values (data deviated within 5% error). Products were quantitatively analysed using an Agilent 7890B gas chromatograph equipped with calibrated thermal conductivity detector (TCD) and flame ionization detector (FID).

2. Supporting figures

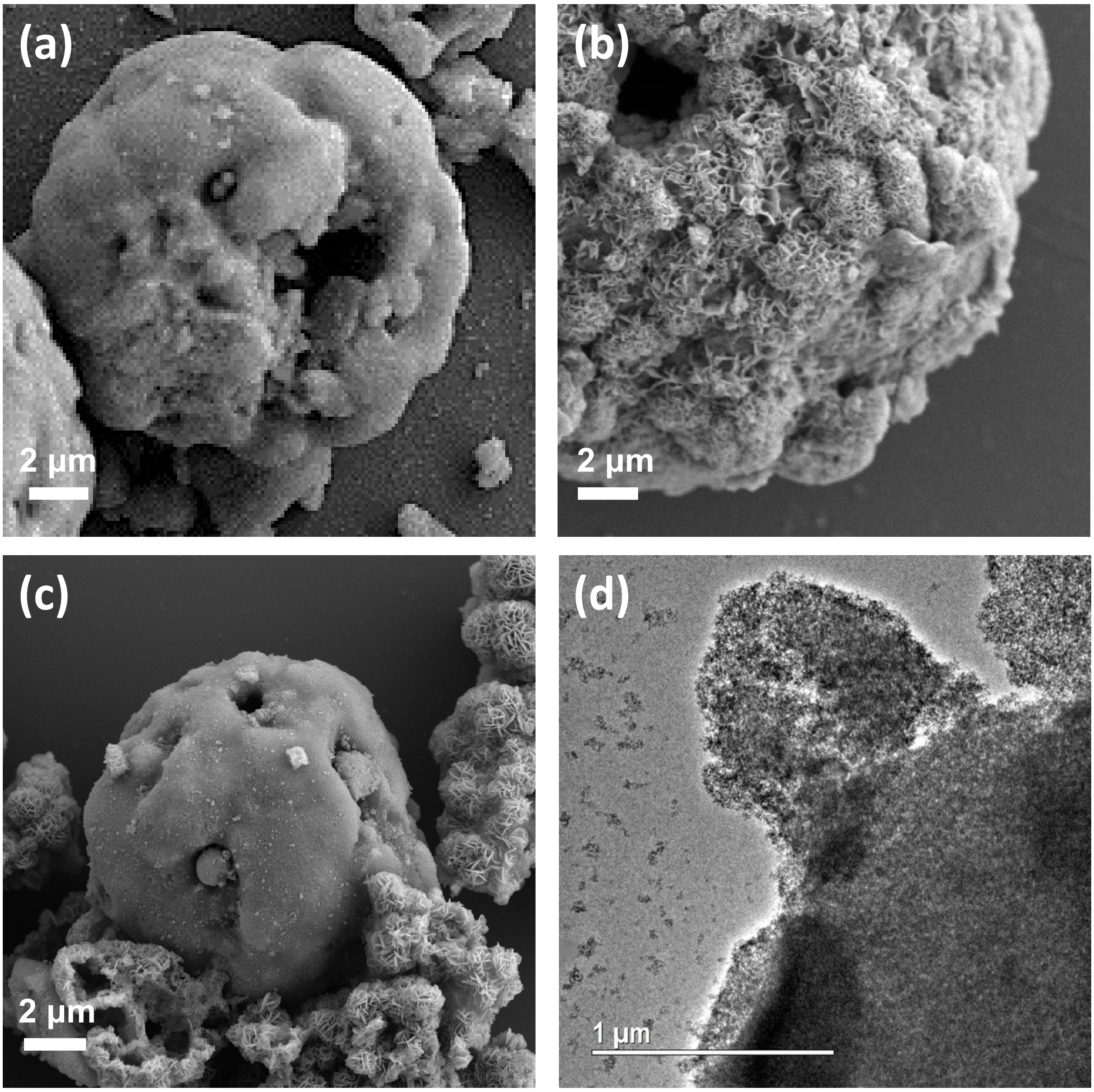

Figure S1. HR-SEM images of (a) ES757, (b) ES757@$Cu_{1.3}ZnAl$ LDH and (c) physical mixture of ES757 and $Cu_{1.3}ZnAl$ LDH; d) TEM images of the physical mixture of ES757 and $Cu_{1.3}ZnAl$ LDH.

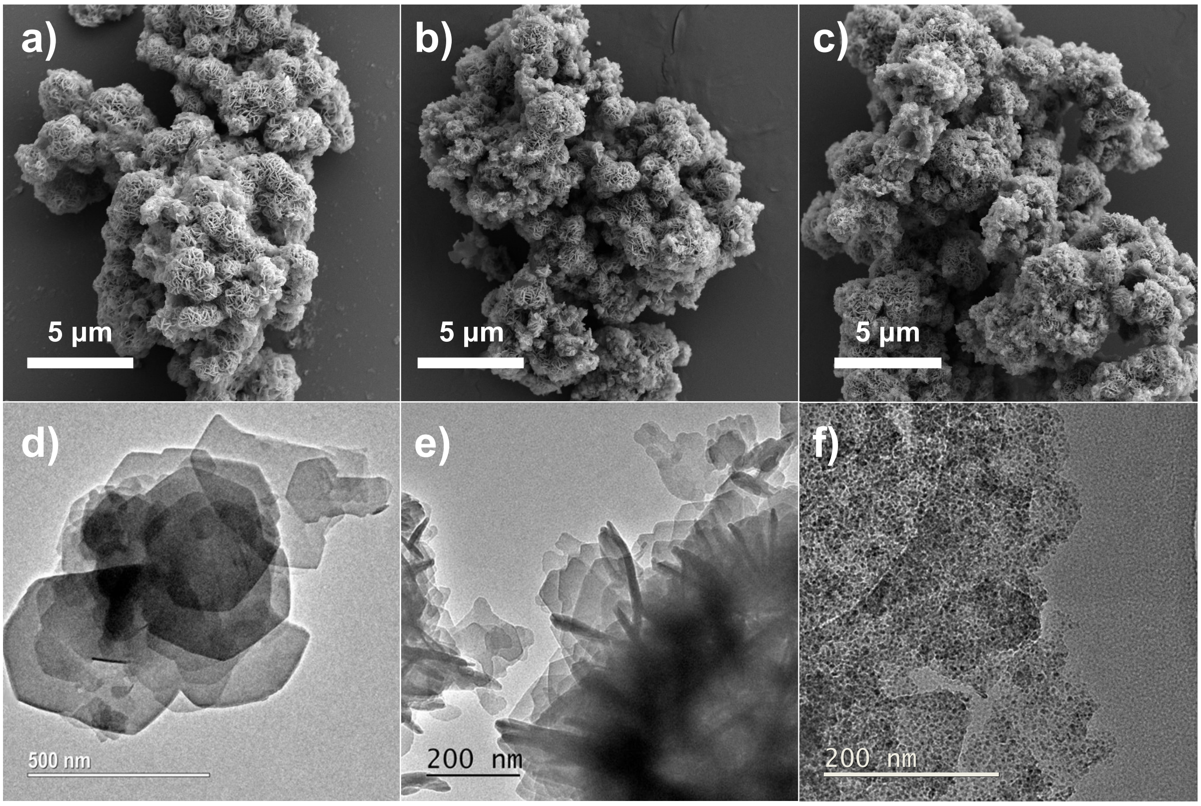

Figure S2. SEM images of a) $Cu_{1.3}$ZnAl LDH; b) $Cu_{1.3}$ZnAl LDO; c) $Cu_{1.3}$. TEM images of d) $Cu_{1.3}$ZnAl LDH; e) $Cu_{1.3}$ZnAl LDO; f) $Cu_{1.3}$.

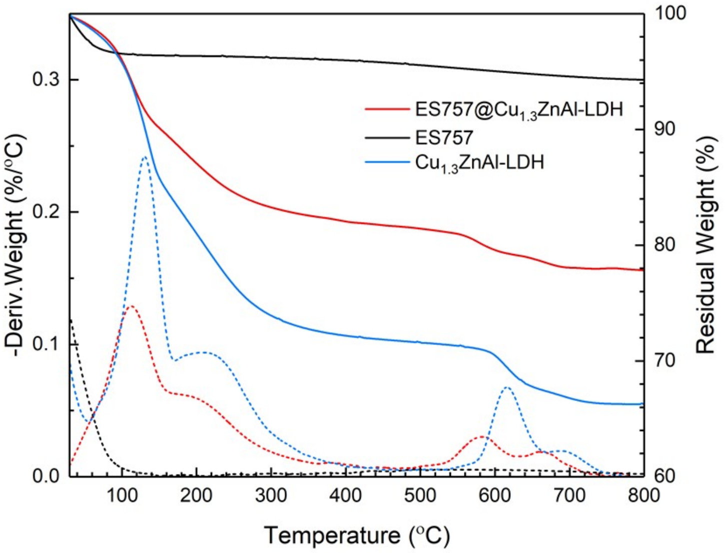

Figure S3. TGA and -Derivative TGA (*d*TG) plots of ES757, ES757@$Cu_{1.3}$ZnAl LDH, $Cu_{1.3}$ZnAl LDH.

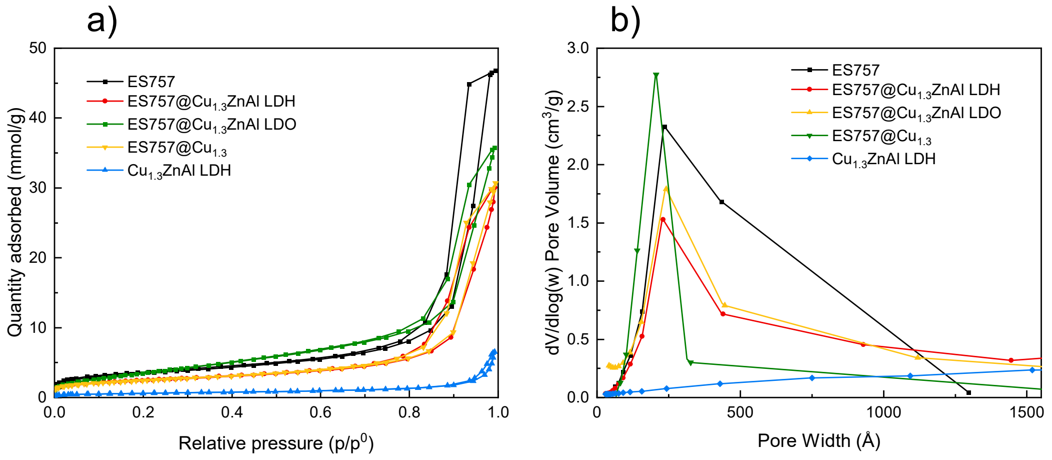

Figure S4. a) N_2 adsorption and desorption isotherm at 77k, b) BJH pore size distribution.

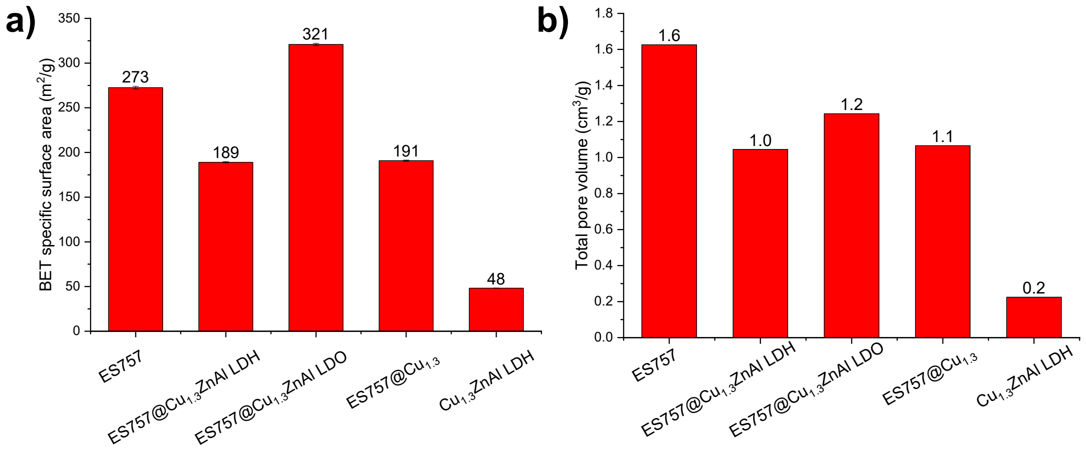

Figure S5. a) The BET specific surface area and b) the total pore volume of ES757, ES757@$Cu_{1.3}$ZnAl LDH, ES757@$Cu_{1.3}$ZnAl LDO, ES757@$Cu_{1.3}$ and $Cu_{1.3}$ZnAl LDH.

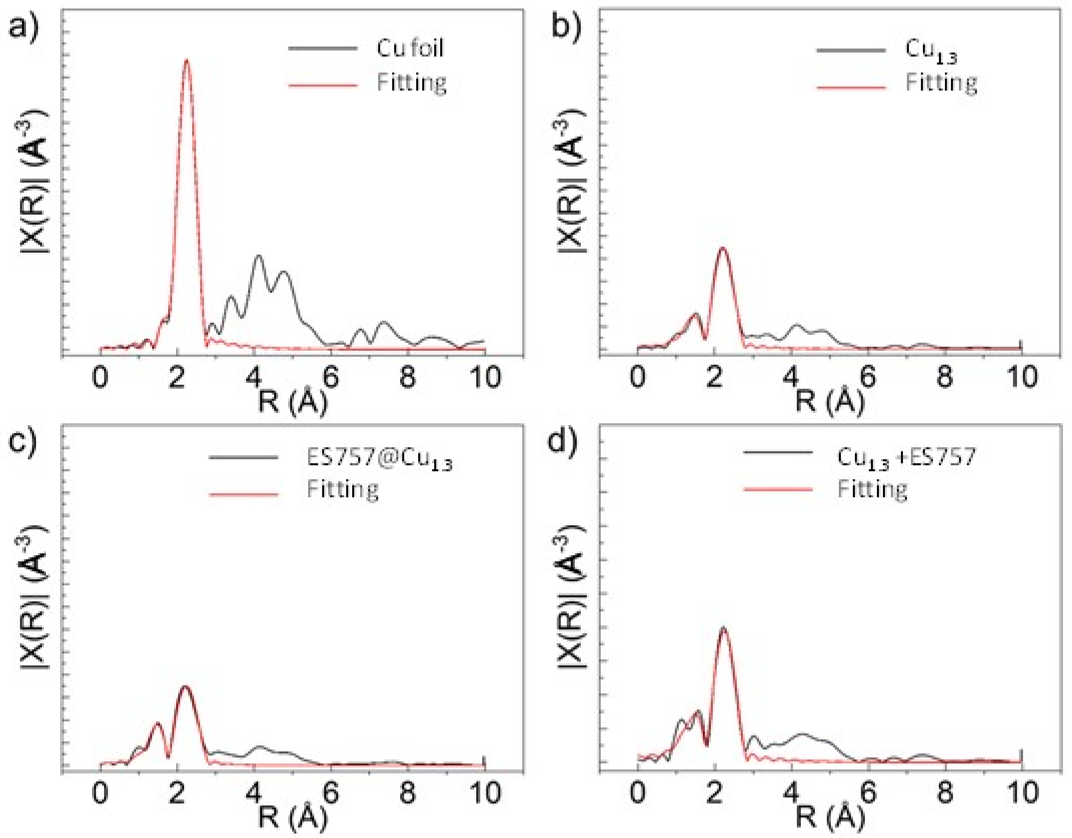

Figure S6. EXAFS fitting of a) Cu foil, b) $Cu_{1.3}$, c) ES757@$Cu_{1.3}$, and d) $Cu_{1.3}$+ES757.

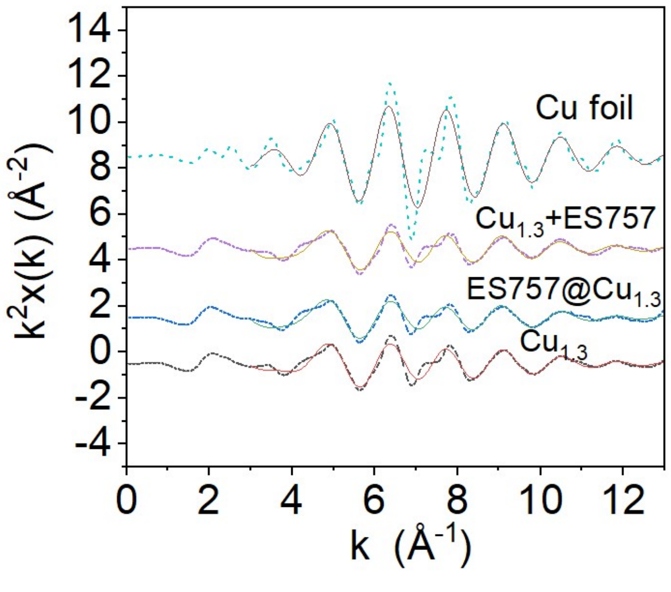

Figure S7. EXAFS fitting to the k-plots.

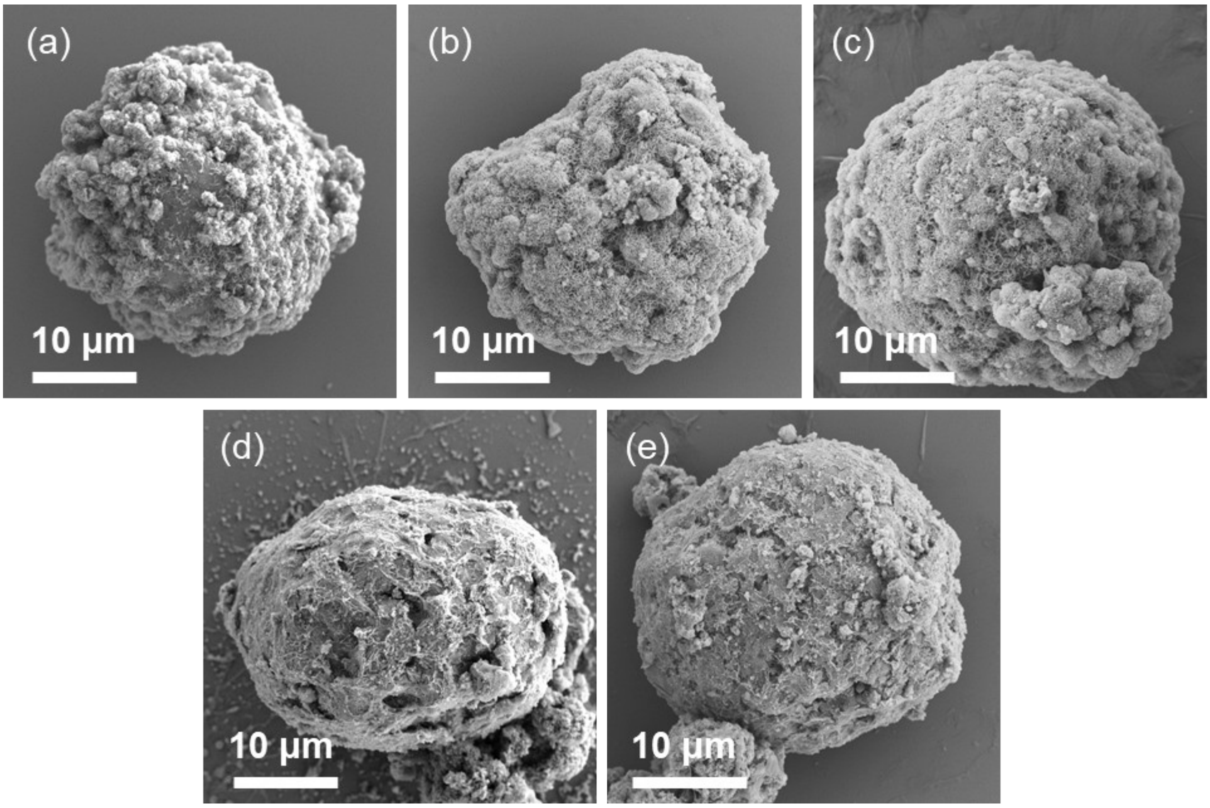

Figure S8. SEM images of (a) ES757@$Cu_{0.8}$ZnAl LDH, (b) ES757@$Cu_{1.3}$ZnAl LDH, (c) ES757@Cu_2ZnAl LDH, (d) ES757@Cu_3ZnAl LDH and (e) ES757@Cu_4ZnAl LDH.

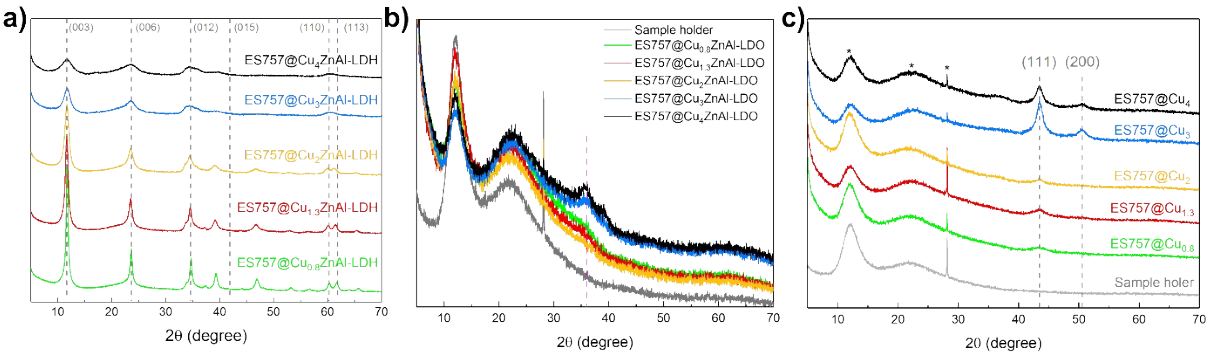

Figure S9. XRD of (a) ES757@Cu_xZnAl LDH, (b) ES757@Cu_xZnAl LDO, (c) ES757@Cu_x.

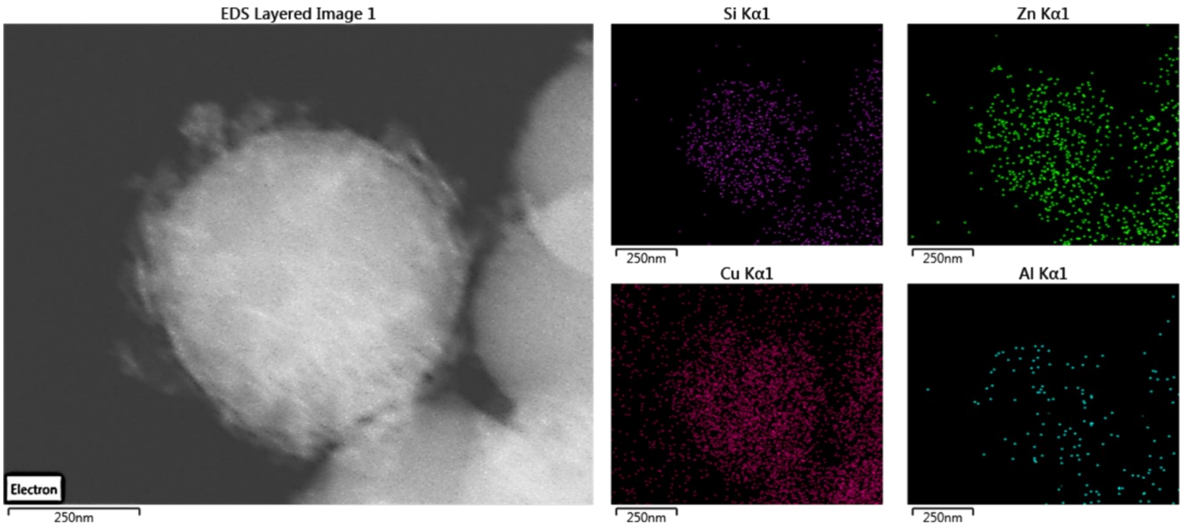

Figure S9. MCM-48@ Cu_2ZnAl LDH and EDX mapping.

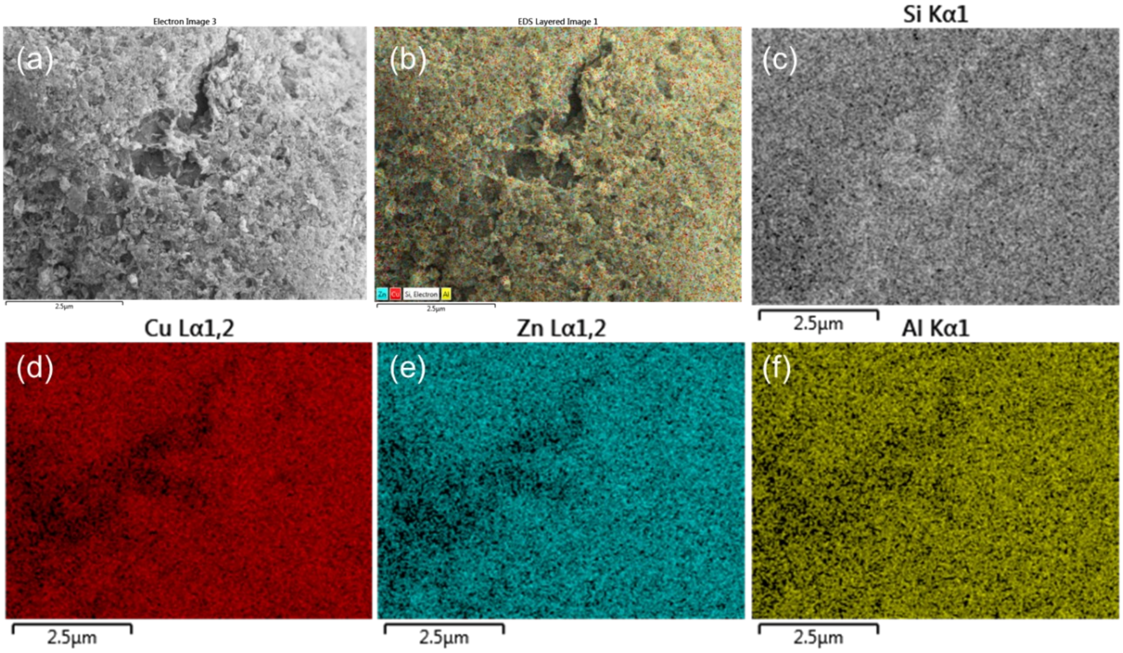

Figure S10 (a) Electron image, (b) elemental mapping layered image, (c)–(f) Si, Cu, Zn and Al mapping of SBA-16@Cu2ZnAl-LDH.

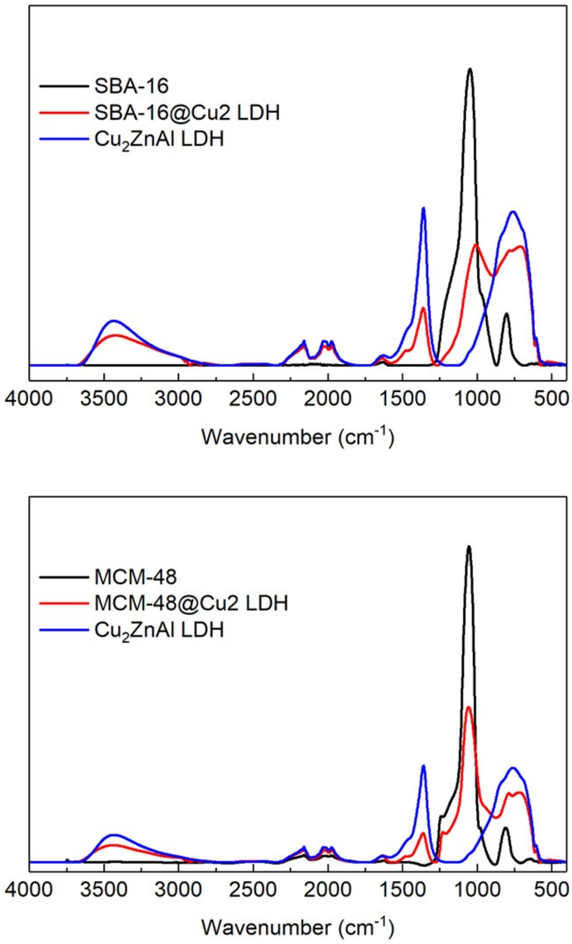

Figure S12. FTIR spectra of SBA-16, MCM-48, their corresponding SiO2@Cu_2ZnAl-LDH core-shells and Cu_2ZnAl-LDH.

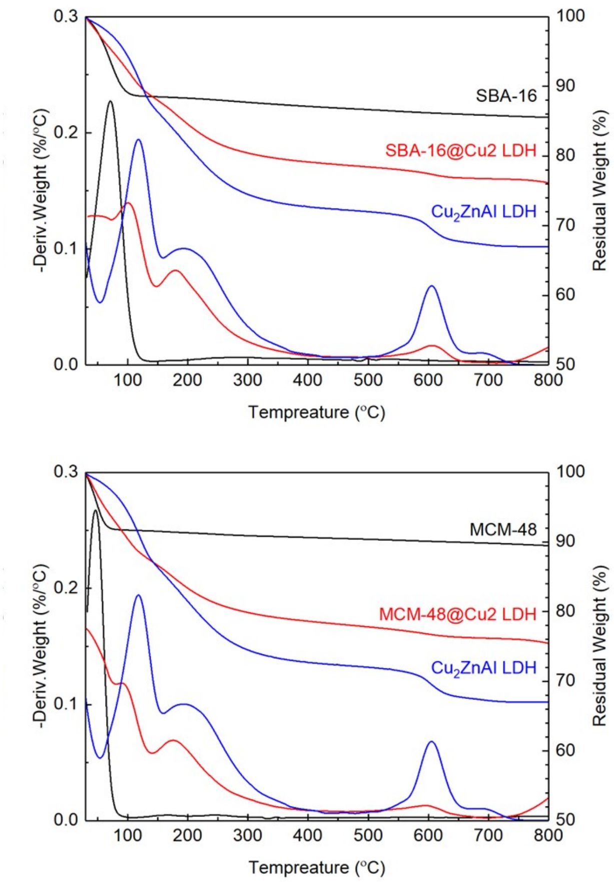

Figure S13. TGA and -dTG of SBA-16, MCM-48, their corresponding SiO2@Cu_2ZnAl-LDH core-shells and Cu_2ZnAl-LDH.

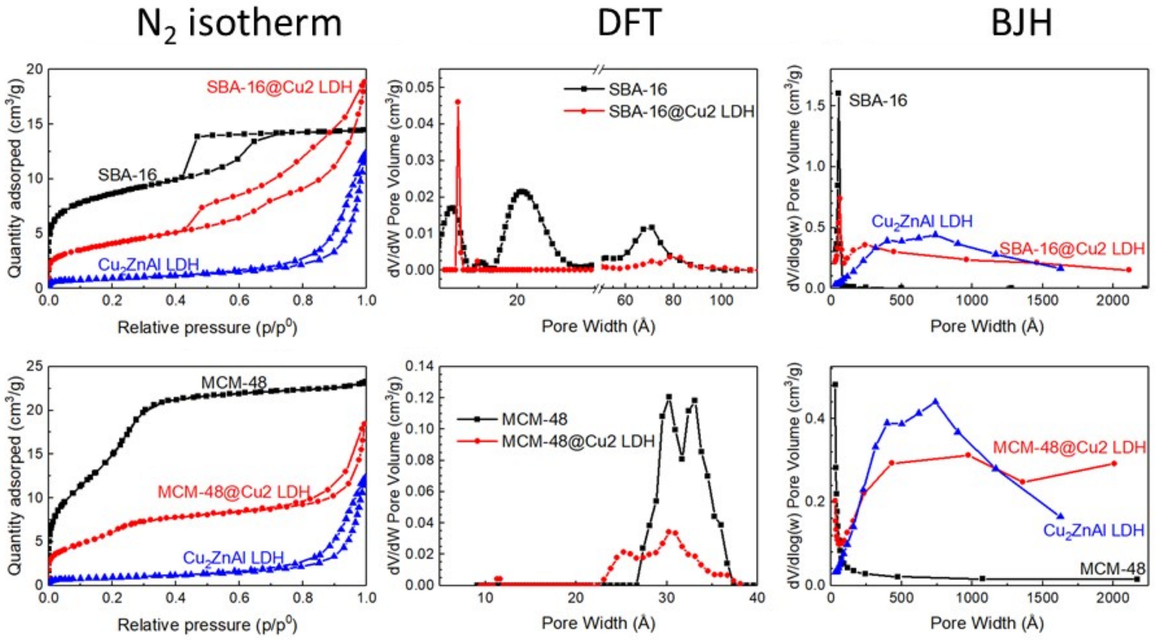

Figure S14. N_2 isotherm, DFT and BJH pore size distribution of SBA-16, MCM-48, their corresponding SiO2@Cu_2ZnAl-LDH core-shells and Cu_2ZnAl-LDH

3. Supporting tables

Table S1. The experimental molar ratio of elements in $Cu_{1.3}$ZnAl LDH, ES757@$Cu_{1.3}$ZnAl LDH, ES757@$Cu_{1.3}$, physical mixture of ES757 and $Cu_{1.3}$ZnAl LDH and the catalyst derived from physical mixture.

	Stoichiometry in LDH $[Cu_aZn_bAl_c(OH)_{2(a+b+c)}(CO_3)_{c/2}]\ nH_2O$						SiO_2
	Cu	Zn	Al	OH	CO_3^{2-}	H_2O	
LDH	1.31	0.93	1.00	6.49	0.49	4.00	0
ES757@$Cu_{1.3}$ZnAl LDH	1.29	1.03	1.00	6.64	0.50	1.49	3.85
ES757@$Cu_{1.3}$	1.43	1.03	1.00	2.05	0.39	-	3.85
$Cu_{1.3}$	1.32	0.96	1.00	1.31	0.35	-	0
Physical mixture	1.34	0.98	1.00	6.64	0.50	1.96	3.85
Physical mixture catalyst	1.33	0.97	1.00	2.61	0.27	-	3.85

Table S2. The Cu loading, Cu dispersion and Cu surface area of ES757@Cu_x catalysts.

Sample	Cu loading (wt%)[a]	Particle size (nm)		Cu dispersion (%)[c]	S_{Cu} ($m^2/g_{catalyst}$)[c]	STY ($g_{MeOH}/g_{catalyst}/h$)
		XRD[b]	TEM			
ES757@$Cu_{0.8}$	15.57	< 3	4.1	17.13	17.32	0.25
ES757@$Cu_{1.3}$	19.81	< 3	3.9	25.00	32.14	0.64
ES757@Cu_2	24.98	< 3	5.7	11.13	18.05	0.43
ES757@Cu_3	32.21	5.79	6.4	9.93	20.76	0.47
ES757@Cu_4	37.40	5.73	7.1	6.98	16.94	0.24
$Cu_{1.3}$+ES757	14.71	-		17.88	17.07	0.42
$Cu_{1.3}$	26.16	-		13.57	23.05	0.34

a: Determined by the ICP-OES. b: Obtained from the Rietveld refinement of the XRD pattern. c: Dispersion and specific surface area of metallic Cu is obtained from N_2O chemisorption results.[43–45]

Table S3. EXAFS fitting parameters.

Sample	Scattering Path	Bond Length (Å)	Coordination number	Debye-Waller Factor (Å^2)	Energy difference (eV)	R factor (%)
Cu foil	Cu-Cu	2.54 (1)	11.7 (0.6)	0.008	5.25	0.37
$Cu_{1.3}$	Cu-Cu	2.54 (1)	5.0 (1.0)	0.010	5.61	2.00
	Cu-O	1.84 (1)	1.2 (0.4)	0.005	5.61	
ES757@$Cu_{1.3}$	Cu-Cu	2.55 (0)	4.1 (0.9)	0.010	6.67	2.20
	Cu-O	1.86 (1)	1.4 (0.4)	0.005	6.67	
$Cu_{1.3}$+ES757	Cu-Cu	2.56 (1)	4.5 (0)	0.010	7.60	2.60
	Cu-O	1.85 (1)	1.7 (0.6)	0.005	7.60	

Table S4. Summary of some best catalysts in the literature including the reaction conditions, CO_2 conversion, MeOH selectivity and STY of methanol. A mixture of H_2/CO_2 (3:1 by molar) gas feed were used in all cases.

Precursor	Reaction temp. (°C)	Reaction press. (bar)	CO_2 conv. (%)	MeOH selec. (%)	MeOH STY ($g_{MeOH}/g_{catalyst}/h$)
ES757@$Cu_{1.3}$ZnAl LDH	270	45	23	48	0.64
MCM-48@Cu_2ZnAl LDH	270	45	21	57	0.70
$Cu_{1.3}$ZnGa AMO-LDH	270	45	18	35	0.59
Commercial Catalyst	270	45	19	49	0.38
Commercial Catalyst[a]	270	45	18	16	0.12

[a]Reduced at 270 ^{o}C.
